# Characterization of spastic paraplegia in a family with a novel *PSEN1* mutation

**DOI:** 10.1093/braincomms/fcad030

**Published:** 2023-02-15

**Authors:** John M Ringman, Naghmeh Dorrani, Sara Gutiérrez Fernández, Rebecca Signer, Julian Martinez-Agosto, Hane Lee, Emilie D Douine, Yuchuan Qiao, Yonggang Shi, Lina D’Orazio, Sanjay Pawar, Leah Robbie, Amir H Kashani, Maxwell Singer, Joshua T Byers, Shino Magaki, Sam Guzman, Abhay Sagare, Berislav Zlokovic, Stephen Cederbaum, Stanley Nelson, Nasim Sheikh-Bahaei, Helena C Chui, Lucía Chávez-Gutiérrez, Harry V Vinters

**Affiliations:** Department of Neurology, Keck School of Medicine at University of Southern California, Los Angeles, CA 90033, USA; Department of Pediatrics, UCLA, Los Angeles, CA 90095, USA; Department of Neurosciences, VIB-KU Leuven Center for Brain & Disease Research, Leuven 3000, Belgium; Department of Neurosciences, Leuven Brain Institute, KU Leuven, Leuven 3000, Belgium; Department of Human Genetics, UCLA, Los Angeles, CA 90095, USA; Department of Human Genetics, UCLA, Los Angeles, CA 90095, USA; Department of Human Genetics, UCLA, Los Angeles, CA 90095, USA; Department of Pathology and Laboratory Medicine, UCLA, Los Angeles, CA 90095, USA; Department of Human Genetics, UCLA, Los Angeles, CA 90095, USA; Department of Neurology, USC Stevens Neuroimaging and Informatics Institute, Los Angeles, CA 90033, USA; Department of Neurology, USC Stevens Neuroimaging and Informatics Institute, Los Angeles, CA 90033, USA; Department of Neurology, Keck School of Medicine at University of Southern California, Los Angeles, CA 90033, USA; Department of Neurology, Keck School of Medicine at University of Southern California, Los Angeles, CA 90033, USA; Department of Neurology, Keck School of Medicine at University of Southern California, Los Angeles, CA 90033, USA; Wilmer Eye Institute, Johns Hopkins University, Baltimore, MD 21287, USA; Roski Eye Institute, Keck School of Medicine, University of Southern California, Los Angeles, CA 90033, USA; Section of Neuropathology, Department of Pathology and Laboratory Medicine, David Geffen School of Medicine, University of California, Los Angeles, CA 90095, USA; Section of Neuropathology, Department of Pathology and Laboratory Medicine, David Geffen School of Medicine, University of California, Los Angeles, CA 90095, USA; Department of Pathology, Keck School of Medicine at USC, Los Angeles, CA 90033, USA; Zilkha Neurogenetics Institute, University of Southern California, Los Angeles, CA 90033, USA; Zilkha Neurogenetics Institute, University of Southern California, Los Angeles, CA 90033, USA; Department of Pediatrics, UCLA, Los Angeles, CA 90095, USA; Department of Human Genetics, UCLA, Los Angeles, CA 90095, USA; Department of Pediatrics, UCLA, Los Angeles, CA 90095, USA; Department of Human Genetics, UCLA, Los Angeles, CA 90095, USA; Department of Pathology and Laboratory Medicine, UCLA, Los Angeles, CA 90095, USA; Department of Radiology, University of Southern California, Los Angeles, CA 90033, USA; Department of Neurology, Keck School of Medicine at University of Southern California, Los Angeles, CA 90033, USA; Department of Neurosciences, VIB-KU Leuven Center for Brain & Disease Research, Leuven 3000, Belgium; Department of Neurosciences, Leuven Brain Institute, KU Leuven, Leuven 3000, Belgium; Section of Neuropathology, Department of Pathology and Laboratory Medicine, David Geffen School of Medicine, University of California, Los Angeles, CA 90095, USA; Department of Neurology, David Geffen School of Medicine, University of California, Los Angeles, CA 90095, USA

**Keywords:** *PSEN1*, spastic paraparesis, F388S, diffusion tensor imaging, flortaucipir

## Abstract

Spastic paraparesis has been described to occur in 13.7% of *PSEN1* mutations and can be the presenting feature in 7.5%. In this paper, we describe a family with a particularly young onset of spastic paraparesis due to a novel mutation in *PSEN1* (F388S). Three affected brothers underwent comprehensive imaging protocols, two underwent ophthalmological evaluations and one underwent neuropathological examination after his death at age 29. Age of onset was consistently at age 23 with spastic paraparesis, dysarthria and bradyphrenia. Pseudobulbar affect followed with progressive gait problems leading to loss of ambulation in the late 20s. Cerebrospinal fluid levels of amyloid-β, tau and phosphorylated tau and florbetaben PET were consistent with Alzheimer’s disease. Flortaucipir PET showed an uptake pattern atypical for Alzheimer’s disease, with disproportionate signal in posterior brain areas. Diffusion tensor imaging showed decreased mean diffusivity in widespread areas of white matter but particularly in areas underlying the peri-Rolandic cortex and in the corticospinal tracts. These changes were more severe than those found in carriers of another *PSEN1* mutation, which can cause spastic paraparesis at a later age (A431E), which were in turn more severe than among persons carrying autosomal dominant Alzheimer’s disease mutations not causing spastic paraparesis. Neuropathological examination confirmed the presence of cotton wool plaques previously described in association with spastic parapresis and pallor and microgliosis in the corticospinal tract with severe amyloid-β pathology in motor cortex but without unequivocal disproportionate neuronal loss or tau pathology. *In vitro* modelling of the effects of the mutation demonstrated increased production of longer length amyloid-β peptides relative to shorter that predicted the young age of onset. In this paper, we provide imaging and neuropathological characterization of an extreme form of spastic paraparesis occurring in association with autosomal dominant Alzheimer’s disease, demonstrating robust diffusion and pathological abnormalities in white matter. That the amyloid-β profiles produced predicted the young age of onset suggests an amyloid-driven aetiology though the link between this and the white matter pathology remains undefined.

## Teaser

Ringman *et al*. describe three affected family members with a novel *PSEN1* mutation with spastic paraparesis with onset at age 23. They describe atypical amyloid-β pathology with imaging and neuropathological evidence of disproportionate effects on corticospinal tracts and found that the F388S mutation causes increased longer length amyloid-β fragments to be produced.

## Graphical Abstract

**Graphical abstract:**
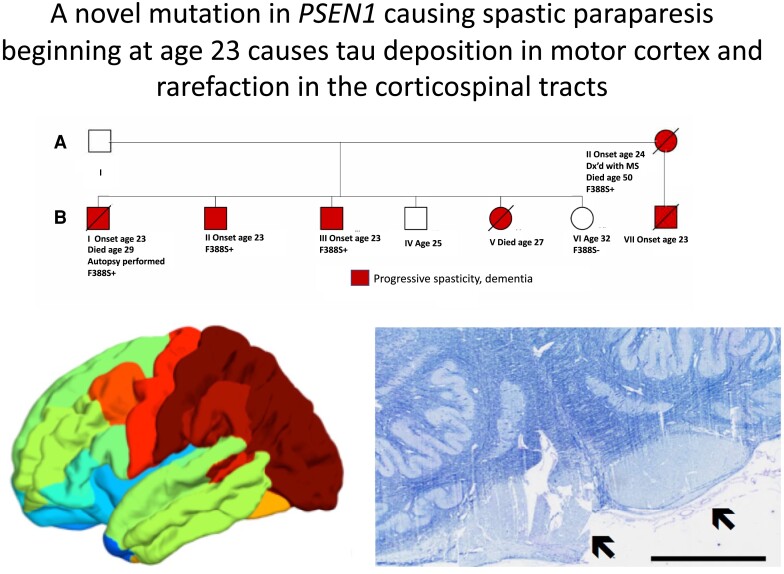

## Introduction

Though autosomal dominant Alzheimer’s disease frequently presents with a progressive amnestic syndrome similar to that seen in late-onset Alzheimer’s disease, it can also present with highly atypical features suggestive of other diagnoses. Atypical presentations including progressive spasticity most evident in the lower extremities (spastic paraparesis or SP) can occur among persons carrying *PSEN1* mutations^1^ and can be considered a form of hereditary spastic paraplegia. Hereditary spastic paraplegia can be classified as uncomplicated (causing paraparesis alone) or complicated (causing other neurological features in addition to paraparesis),^1^ and since SP can be the earliest manifestation of *PSEN1*-related Alzheimer’s disease, *PSEN1* mutations should be considered in the differential diagnosis of apparent inherited myelopathies.^2,3^

Among the genes causing autosomal dominant Alzheimer’s disease, age of disease onset is youngest among carriers of *PSEN1* mutations^4^ and tends to be consistent within autosomal dominant Alzheimer’s disease mutations but can vary between them.^5^ The reasons underlying the variation in phenotype and onset age among diverse *PSEN1* mutations are not completely defined. Differences in the qualitative and quantitative nature of γ-secretase cleavage, leading to production of differential amounts of amyloid-β (Aβ) species of variable lengths,^6^ potentially through altered kinetics of its interaction with amyloid precursor protein (APP)^7^ likely play a role. A recent paper demonstrated a linear relationship between the ratio of shorter (Aβ37, Aβ38 and Aβ40) to longer (Aβ42 and Aβ43) length Aβ species and age of symptom onset among 25 autosomal dominant Alzheimer’s disease mutations.^2^ The association of amyloid plaques with unusual composition and morphology (i.e. ‘cotton wool’ plaques) with *PSEN1* mutations and SP is consistent with diverse profiles of Aβ species playing a role.^1^ However, contributions to phenotypic variability from differences in γ-secretase cleavage of other substrates^8^ or other, non-γ-secretase functions of presenilin-1 cannot be excluded.^9^ As a microarray analysis of brain expression data showed co-expression of *PSEN1* with multiple myelin-associated genes^10^ and SP in *PSEN1*-related autosomal dominant Alzheimer’s disease has been demonstrated to be associated with widespread white matter abnormalities,^11^ a more direct effect of these mutations on cerebral connectivity not mediated by APP processing should be considered. The relationship of pathogenic *PSEN1* mutations and white matter integrity is therefore of interest.

Here, we describe the phenotype, imaging, ophthalmological and neuropathological features of a family with onset of progressive debilitating spastic paraplegia at age 23 due to a novel mutation in the *PSEN1* gene. We then relate diffusion imaging and flortaucipir PET characteristics of three affected family members to those with other autosomal dominant Alzheimer’s disease mutations with and without SP. Finally, we assess the relationship of the pattern of Aβ species resulting from this mutation in an *in vitro* assay to the age of symptom onset.

## Materials and methods

### Description of cases

**Figure 1:**
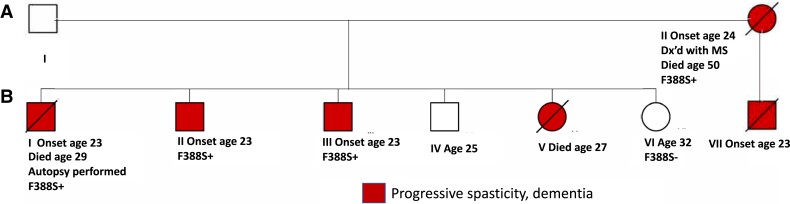
**Pedigree showing affected persons and demonstrating co-segregation of the F388S *PSEN1* substitution with the disease.** All family members presented at age 23 or 24 with difficulty walking due to leg stiffness progressing to being wheelchair bound with diffuse spasticity, dysarthria and dementia.

The index patient (patient A2 in the pedigree, Fig. 1) was a Mestizo woman from El Salvador who began having problems with walking and falls at age 24. Her symptoms progressed to include dementia and dysphagia and she was diagnosed with multiple sclerosis. She was ultimately bed bound in her late 20s, placed on a feeding tube, a ventilator and ultimately died in her mid-50s.

The medical history of her parents is unknown. She had seven children, one from one father and six from another. The son from the first father (BVII) began to have problems walking at age 23, which progressed to his requiring a walker and then a wheelchair. He was described as having extreme muscular tightness in his arms and legs. He developed problems with speech production and was non-verbal though was thought to recognize familiar faces until his death at age 34. Of the six children from the other father, one daughter (BV) died at age 27 with progressive paraplegia, a son had leg stiffness and an unsteady gait beginning at age 23 (BI), and two other sons began having leg stiffness at age 23 (BII and BIII). A son aged 28 (BIV) is described as healthy, and a daughter is healthy at age 32 (BVI).

The affected brothers BI, BII and BIII underwent comprehensive evaluations. Brother BI had onset of leg stiffness and difficulty walking at age 23, followed by problems with executive function at age 26. When examined at age 28, he was bed bound with dysarthria and pseudobulbar affect. He had spasticity of his arms and legs (Ashworth score of 4^12^), elevated deep tendon reflexes in his lower (4+) and upper (3+) extremities. Babinski and palmomental reflexes were present.

Brother BII was healthy until he began having difficulty walking due to leg stiffness at age 23, followed by cognitive decline at age 24. When seen at age 25, he had dysarthria and could walk independently with difficulty using a walker. Tone was slightly elevated in his arms though markedly so in his legs, being 4 on the Ashworth Scale. Deep tendon reflexes were 3+ in his upper extremities and Achilles’ tendons bilaterally and 4+ at the patellae. Babinski signs were present. His Clinical Dementia Rating (CDR) score was 1.0 with a sum of boxes score of 6.5.

He scored 23/30 on the Mini-Mental Status Examination.^13^ Results from formal neuropsychological testing revealed deficits across all domains assessed. Specifically, executive functions that were impaired included attention, working memory and processing speed; however, complex mental tracking (Trails B) was in the average range. Performance on measures of rote and contextual memory, visual memory, visuospatial construction, confrontation naming and verbal fluency (semantic and phonemic) were impaired.

When seen again 14 months later, he had had significant progression of his spasticity, needing assistance to walk. Though he initially presented without pseudobulbar affect, this was apparent at the subsequent visit. Follow-up neuropsychological assessment revealed slight improvements in attention (borderline impaired), working memory (low average), but complex mental tracking declined significantly to the impaired range. Additionally, speech fluency declined further from the initial assessment. Though he had significant cognitive slowing and difficulty communicating verbally due to dysarthria, he was still making his appointments independently via text message. His CDR score was still 1 and sum of boxes score was 7.5.

Brother BIII is a healthy man who began developing stiffness of his gait and executive dysfunction at age 23. When seen at age 25, he was ambulatory with 3/4 rigidity in his right, 2/4 rigidity in his left lower and 1/4 rigidity in his upper extremities (by Ashworth score). Deep tendon reflexes were 3+ in the upper and 4+ in the lower extremities, and Babinski signs were present bilaterally. Speech was slightly dysarthric and pseudobulbar affect was apparent. He scored 19/30 on the Montreal Cognitive Assessment and formal neuropsychological testing revealed deficits, in descending order of severity, in executive function, memory, language and visuospatial function. He demonstrated significant depression and anxiety as well. His CDR score was 0.5 with a sum of boxes score of 1.0. He was thought to have amnestic MCI, affecting multiple domains.

Imaging parameters were compared between the two brothers with the F388S mutation, seven persons with the A431E *PSEN1* mutation and three persons with autosomal dominant Alzheimer’s disease mutation not causing SP matched for disease severity using the CDR scale.^14^ The Ashworth score for leg spasticity (scores ranging from 0 to 4) was rated for all participants seen in person.^12^

### Genetic evaluations

Family member BI underwent comprehensive genetic assessment. A panel for trinucleotide repeats for spinocerebellar atrophies was performed at MNG laboratories. Exome sequencing was also performed as a duo, with the index mother (A2) at Invitae. Whole-genome sequencing was performed as a trio with the index mother (A2) and the 29-year-old unaffected sister (BVI) at UCLA’s California Center for Rare Diseases as previously described.^15^ Targeted sequencing was performed on family members BII and BVI at the UCLA Orphan Disease Testing Center.

### Imaging methods

All participants including the affected brothers BI (age 28) and BII (age 25) underwent MRI using the Human Connectome Protocol and PET for tau pathology using flortaucipir. Brother BII also underwent amyloid PET imaging using florbetapir at age 27.

For tau PET, 10.3 mCi 18F-AV-1451 was administrated through an intravenous catheter and images were obtained beginning 75 min after injection. Six frames, 5 min apart, were collected and averaged. A low-dose CT transmission scan was obtained for attenuation correction. For amyloid PET, ∼50 min after intravenous administration of 10.4 mCi of ^18^F-florbetapir, PET images were obtained of the brain from vertex to skull base. Low-dose CT scan was obtained over the same anatomic range for attenuation correction.

To quantify flortaucipir PET imaging, we first ran recon-all script from FreeSurfer 6.0 to get high-resolution segmentations from the T1 image. Desikan–Killiany atlas^16^ was used to define 36 anatomical regions. PET images were then co-registered to T1 native space. The Muller-Gartner method was used to correct the partial volume effect of flortaucipir PET image using PETSurfer in FreeSurfer 6.0. Standard uptake value ratios (SUVRs) were then calculated using cerebellar grey matter from the T1 image as the reference region. Partial volume–corrected SUVR was also mapped to the cortical surface that is parcellated to 36 regions for each cerebral hemisphere.

In order to investigate the integrity of white matter associated with the F388S *PSEN1* substitution, diffusion MRI was performed using the Human Connectome Protocol. T1-weighted MR image and diffusion MR image of each subject were preprocessed by Human Connectome Protocol pipeline^17^ with version 3.27. Diffusion MRI data were acquired with two opposite phase encoding directions that are anterior to posterior (AP) and posterior to anterior (PA). Raw diffusion MRI data were corrected by topup and eddy functions in FSL to reduce the distortion caused by susceptibility-induced distortion and eddy current-induced distortion. After distortion correction, diffusion MRI data were resampled to T1 image space. The diffusion tensor model and associated eigenvalues (*λ*_1_, *λ*_2_, *λ*_3_) were estimated with the MRtrix3 software.^18^ Fractional anisotropy, mean diffusivity (MD), radial diffusivity and axial diffusivity were then computed. These diffusivity measures were mapped back to T1 space using the linear transformation obtained from the registration between B0 image and T1 image processed by recon-all. White matter is also parcellated into 36 regions for each cerebral hemisphere using the method as cortical region parcellation. Mean values of these diffusivity measures in each white matter region are then calculated for statistical analysis.

### Cerebrospinal fluid analysis

#### Amyloid-β peptide

MSD assay (catalog no. K15199G, MSD, Rockville, MD) was used to determine CSF levels of Aβ38, 40 and 42.

#### Tau

MSD assay (catalog no. K15121G, MSD, Rockville, MD) was used to determine CSF levels of total tau. Phosphorylated tau (pT181) was determined by ELISA (catalog no. 81581, Innotest, Belgium).

### Ophthalmic examination and imaging methods

Brothers BII and BIII underwent a complete ophthalmic examination by a board-certified ophthalmologist. Ancillary testing including colour fundus photography and optical coherence tomography (OCT) of the macula was performed to document the appearance of the retina and any qualitative retinal findings.

### Neuropathologic methods

An autopsy restricted to the brain (including a small portion of cervical spinal cord) was performed on subject BI. The brain was extensively sampled according to the UCLA dementia protocol including representative sections from the frontal, temporal, parietal and occipital cortices, hippocampus, entorhinal cortex, amygdala, basal ganglia, brainstem and cerebellum. Six-micrometre sections were cut from formalin-fixed paraffin-embedded tissue and were stained with haematoxylin and eosin. Several blocks were also stained with Luxol fast blue. Immunohistochemistry was implemented with antibodies to β-amyloid 1–42 (1:150, EMD Millipore, rabbit polyclonal, AB5078P), β-amyloid 1–40 (1:400, EMD Millipore, rabbit polyclonal, AB5074P), phospho-tau (1:200, Thermo Fisher, mouse monoclonal, AT8) and alpha-synuclein (1:450, EMD Millipore, rabbit polyclonal, AB5038). Incubation with primary antibodies was followed by either horse anti-mouse or horse anti-rabbit secondary antibody conjugated to horseradish peroxidase (MP7402 & MP7401; Vector Laboratories, Burlingame, CA, USA). Visualization of antibody reactivity was achieved with *N*′*N*-diaminobenzidine as chromogen (no. SK-4100; Vector Laboratories) and then counterstained with haematoxylin. Neuropathologic substrates of dementia were assessed using standard diagnostic criteria.^19^ The presence of cerebrovascular disease was also evaluated, including cerebral amyloid angiopathy (CAA) graded according to the Vonsattel criteria.^20^

All three participants with the F388S mutation, 5/8 of the A431E mutation carriers and 2/3 carriers of *PSEN1* mutations not causing SP were male. We therefore were unable to assess effects of gender.

### Amyloid-β profile assay

The expression of *PSEN1* [wild type (WT) or F388S mutant] was rescued in *PSEN*-deficient mouse embryonic fibroblasts using the replication-defective pMSCV retroviral expression system (Clontech), following protocols previously described.^21^ Briefly, the F388S mutation was introduced in the cDNA encoding for human PSEN1 using the Q5 mutagenesis kit (New England BioLabs) and the following mutagenic primers: forward: 5′-AGATTTCATTtccTACAGTGTTCTG-3′; reverse: 5′-CCCAATCCAAGTTTTACTC-3′. Recombinant retroviruses were generated by co-transfection of human embryonic kidney (HEK293T17) cells with the pMSCVpuro retroviral vector (bearing the cDNA encoding for WT or mutant F388S *PSEN1*) and a helper plasmid containing genes necessary for viral packaging. Retroviral particles, harvested at 48 h post-transfection, were used in the transduction of Psen1-l-/Psen2-l- mouse embryonic fibroblasts, and clones stably expressing (WT or mutant) PSEN1 were selected in DMEM/F-12 medium supplemented with 10% foetal bovine serum and puromycin (5 µg/ml). Reconstitution of mature γ-secretase complexes was verified in the WT and mutant cell lines by analysis of nicastrin, *PSEN1* C-terminal fragment, and PEN-2 protein levels in SDS-PAGE/western blotting. Western Lightning Plus-ECL Enhanced Chemiluminescence Substrate (PerkinElmer) and Fuji imager were used.

To determine the effect of the F388S *PSEN1* mutation on APP processing, WT- and F388S *PSEN1* cell lines were transduced with recombinant adenoviruses encoding for human APPC99 and green fluorescence protein. Green fluorescence protein expression reported on transduction efficiency. At 16 h post-transduction, media was exchanged (DMEM/F-12, supplemented with 0.2% foetal bovine serum), and after 24 h (∼30 h post-transduction), condition medium samples were collected for Aβ profile analysis. Secreted Aβ37, Aβ38, Aβ40, Aβ42 and Aβ43 peptide levels were measured in conditioned medium samples, using multi-Spot 96-well MSD ELISA (simultaneous quantification of Aβ37, Aβ38, Aβ40 and Aβ42) and Aβ43 ELISA (IBL), as reported.^21^

### Statistical analysis

ANOVA’s comparing clinical features, flortaucipir PET SUVRs and MD from diffusion tensor imaging (DTI) among the three brothers with the F388S *PSEN1* mutation, eight persons with the A431E *PSEN1* mutation (seven for flortaucipir PET) and three persons with autosomal dominant Alzheimer’s disease mutations not causing SP were performed using Statistical Package for the Social Sciences, version 27.

### Data availability

De-identified data are available upon request.

## Results

### Genetic testing results

As the proband BI was diagnosed with hereditary SP, a panel for trinucleotide repeats causing spinocerebellar atrophies was performed and was negative. Through exome sequencing, he was found to have a single heterozygous Ala510Val variant in *SPG7*, which, when present in the homozygous state, can cause SP. This variant was not found in the similarly affected mother. Then, trio whole genome sequencing with the index mother (A2) and the 29-year-old unaffected sister (BVI) revealed a novel F388S substitution in *PSEN1* (cDNA NM_000021.3:c1163T > C) that co-segregated with the disease. Codon 388 in *PSEN1* is adjacent to the site of one of the aspartate residues critical for γ-secretase activity (codon 385). The F388S substitution is predicted by Polyphen to be ‘probably pathogenic’ (http://genetics.bwh.harvard.edu/pph2/). Genomic DNA of family members BII and BVI was subjected to targeted sequencing and identified one copy (heterozygous) of the *PSEN1* variant c.1163T > C in affected member BII.

### Imaging results

**Figure 2:**
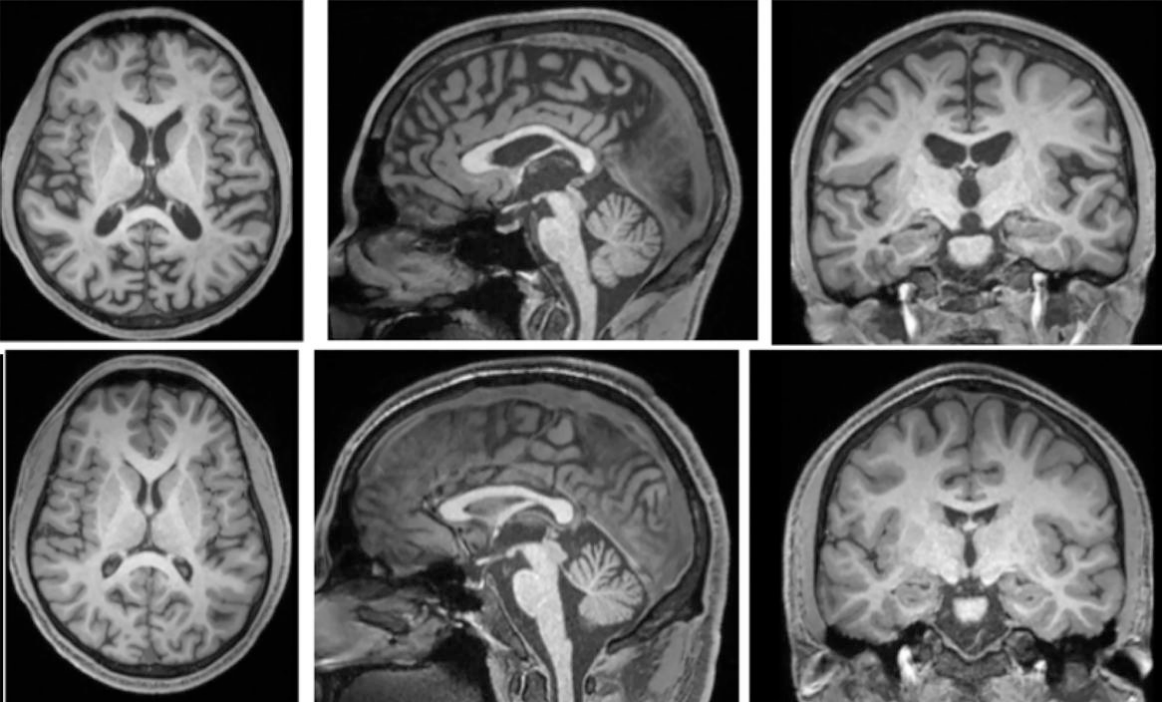
**Structural T1-weighted MRIs showing affected brothers BI (top row) at age 28 and BII (bottom row) at age 25.** Mild atrophy is present only in the older brother with more advanced disease; atrophy was still minimal at autopsy at age 29.

**Figure 3:**
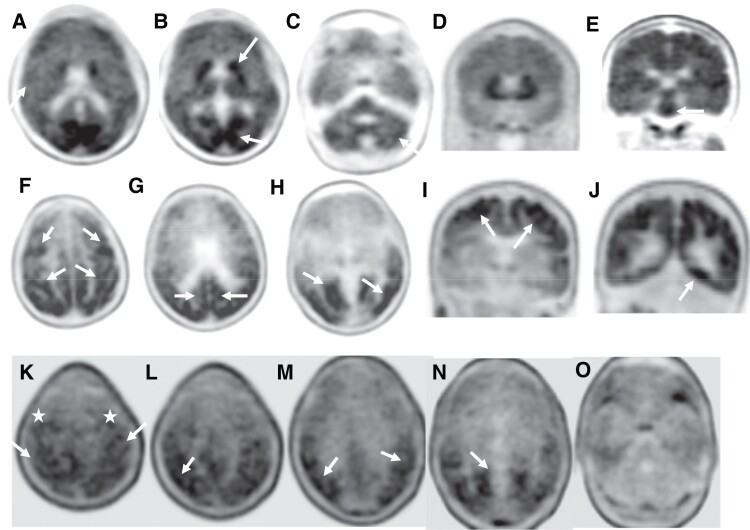
**Amyloid and tau PET images in affected family members.** (1. *Top* row) Florbetapir PET images of Case BII in axial (**A**–**C**) and coronal (**D** and **E**) planes showing loss of grey–white matter differentiation (**A**, arrow) with multiple areas of high cortical uptake particularly in the posterior cingulate gyri, parietal and occipital lobes with very high uptake in the basal ganglia and brainstem (**B** and **E**, arrows). Patchy uptake is also seen in the grey matter of the cerebellum (**C**, arrow). (2*. Middle* row) Flortaucipir PET images of case BI; axial (**F**–**H**) and coronal (**I** and **J**) planes show extensive tau uptake in the neocortical regions. There are bilateral frontal and parietal uptakes including the peri-Rolandic regions (**F** and **I**, arrows), posterior cingulate gyri (**G**, arrow) and premotor cortices (**F**, top arrows). There is also extensive binding in the lateral and medial occipital areas bilaterally (**G** and **J**, arrows). (3. *Bottom* row) Flortaucipir PET-CT images of case BII; axial images showing patchy uptake in neocortical regions, including parietal, occipital, posterolateral temporal lobes (**L–O**, arrows) and also in the peri-Rolandic regions (**K**, arrows) and premotor cortices (**K**, white stars). No cerebellar uptake identified.

T1-weighted structural MRI on the affected brothers showed only mild diffuse atrophy most evident in the brainstem in BI at age 28 years (Fig. 2). The florbetapir PET images of case BII (Fig. 3A–E) show widespread loss of grey–white matter differentiation in several cortical regions. Additionally, there are multiple regions of dense high cortical uptake above the level of uptake in the underlying white matter, particularly in the posterior cingulate gyri, in the occipital lobes and, to a lesser degree, in the frontal lobes in keeping with high level of amyloid deposition. Moreover, there is marked uptake in the basal ganglia including caudate, lentiform nuclei and thalami with extension through the corticospinal tracts into the midbrain and pons that have milder uptake. There is also patchy uptake in the cerebellar grey matter bilaterally (Fig. 3C).

The flortaucipir PET study of case BII (Fig. 3K–O) demonstrates neocortical uptake in several regions, particularly in the occipital, posterolateral temporal, parietal lobes and to a lesser degree in the frontal regions involving the peri-Rolandic area and premotor cortex presenting an ‘advanced Alzheimer’s disease tau pattern’. Flortaucipir PET in case BI (Fig. 3F–J) also shows widespread neocortical uptake with marked binding in occipital, posterolateral temporal, parietal, posterior cingulate gyri, peri-Rolandic regions and premotor cortex with milder uptake in the remaining frontal lobes, also in keeping with an ‘advanced Alzheimer’s disease tau pattern’.

**Figure 4:**
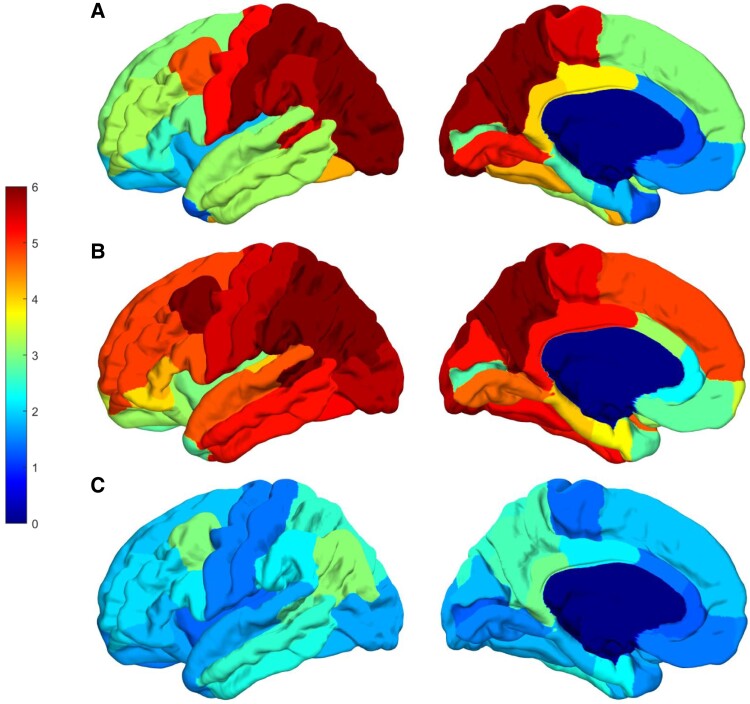
**SUVRs for flortaucipir PET in persons affected by autosomal dominant Alzheimer’s disease mutations.** The averaged SUVR for both cerebral hemispheres shown on the left hemisphere of the fsaverage template from all subjects in each group. For each subject, SUVR value was first averaged for each region of interest and then left and right hemispheres were averaged shown here on the fsaverage template for each group. The *top* row (**A**) is data from the two brothers affected by the F388S mutation, the *middle* row (**B**) is derived from seven persons affected by the A431E mutation in *PSEN1*, and the *bottom* row (**C**) is derived from three persons affected by autosomal dominant Alzheimer’s disease mutations that do not cause spastic paraparesis. Note the variable involvement of the Rolandic cortex associated with presence or absence of SP but consistent involvement of the precuneus across groups.

We performed quantitative comparisons of flortaucipir SUVR’s and DTI measures among the brothers with the F388S *PSEN1* mutation (*n* = 2), carriers of the A431E *PSEN1* mutation that features SP though at a more advanced age,^22, 23^ matched for overall disease severity by CDR sum of boxes score (*n* = 8 for DTI measures, 7 for tau PET measures) and carriers of other autosomal dominant Alzheimer’s disease mutations that do not feature SP (*n* = 3). The carriers of the F388S mutation had Ashworth scores of 4, 4 and 2–3, while those with the A431E mutation had scores of 0, 1 (#3), 2 (#2), 3 and 4. One-way ANOVAs suggested increased signal in the peri-Rolandic cortex and in the pericalcarine areas among carriers of mutations that cause SP relative to those with mutations that do not, with those having the F388S mutation and most severe spasticity having the numerically greatest signal in the paracentral lobule, where the corticospinal tract subserving the lower extremities originates. However, these results did not reach statistical significance (Table 1, Fig. 4).

**Table 1:** ANOVA’s comparing clinical features, flortaucipir PET SUVRs and MD from DTI among the three brothers with the F388S *PSEN1* mutation, eight persons with the A431E *PSEN1* mutation (seven for flortaucipir PET) and three persons with autosomal dominant Alzheimer’s disease mutations not causing SP

	Persons with the F388S *PSEN1* mutation (*n* = 3)	Persons with the A431E *PSEN1* mutation (*n* = 8, 7 for tau PET)	Persons with autosomal dominant Alzheimer’s disease mutations not causing SP (*n* = 3)	
Age in years	25.7 (1.24)	42.4 (3.3)	56.7 (11.4)	***P*** ***<*** 0.001*
Gender (# male)	3	5	2	
CDR sum of boxes	7.5 (7.1)	8.81 (4.27)	3.67 (2.52)	*P* = 0.348
Ashworth score in legs,	3.5 (0.87)	1.75 (1.28)	0.0 (0.0)	***P*** **=** 0.008*
SUVR, entorhinal cortex	1.60 (0.51)	3.85 (1.46)	2.19 (0.92)	*P* = 0.045
SUVR, precuneus	5.41 (3.94)	6.63 (3.20)	2.78 (1.95)	*P* = 0.227
SUVR, precentral gyrus	4.29 (3.24)	5.50 (4.11)	1.50 (0.58)	*P* = 0.299
SUVR, paracentral gyrus	4.52 (3.38)	5.18 (3.51)	1.33 (0.22)	*P* = 0.244
SUVR, pericalcarine cortex	2.51 (1.65)	2.70 (1.44)	1.29 (0.18)	*P* = 0.340
MD, corpus callosum	0.00088 (0.000049)	0.06887 (0.013520)	0.013520 (0.022087)	*P* = 0.761
MD, left CST	0.000808 (0.000059)	0.000719 (0.000040)	0.000683 (0.000021)	***P*** **=** 0.008*
MD, right CST	0.000811 (0.000048)	0.000733 (0.000033)	0.000680 (0.000030)	***P*** **=** 0.003*
MD, left precentral gyrus	0.000676 (0.000068)	0.000534 (0.000030)	0.000498 (0.000020)	***P*** ***<*** 0.001*
MD, right precentral gyrus	0.000676 (0.000072)	0.000538 (0.000014)	0.000511 (0.000024)	***P*** ***<*** 0.001*
MD, left paracentral lobule	0.000683 (0.000077)	0.000542 (0.000032)	0.000507 (0.000031)	***P*** **=** 0.001*
MD, right paracentral lobule	0.000683 (0.000065)	0.000556 (0.000027)	0.000503 (0.000029)	***P*** ***<*** 0.001*
MD, left entorhinal cortex	0.000693 (0.000087)	0.000594 (0.000037)	0.000593 (0.000022)	*P* = 0.038
MD, right entorhinal cortex	0.000691 (0.000091)	0.000600 (0.000035)	0.000611 (0.000013)	*P* = 0.051
MD, left precuneus	0.000711 (0.000089)	0.000592 (0.000033)	0.000542 (0.000032)	***P*** **=** 0.005*
MD, right precuneus	0.000701 (0.000079)	0.000602 (0.000024)	0.000540 (0.000030)	***P*** = 0.003*
MD, left pericalcarine	0.000737 (0.000093)	0.000607 (0.000017)	0.000570 (0.000040)	***P*** **=** 0.003*
MD, right pericalcarine	0.000713 (0.000068)	0.000604 (0.000022)	0.000580 (0.000040)	***P*** **=** 0.003*

Measures in parentheses are standard deviations and bold-faced *P*-values (*) are those less than 0.01. Note that SUVRs are averages between the two hemispheres.

One-way ANOVAs comparing DTI measures were performed among the same groups defined above. There was an overall pattern of increased MD among carriers of mutations causing SP across all white matter regions with the brothers with the F388S mutation having the highest values, those with the A431E mutation having intermediate values and those with mutations not causing SP having the lowest values. These differences were most profound beneath the peri-Rolandic cortex and in the corticospinal tracts and corpus callosum (Table 1).

### Cerebrospinal fluid findings

A lumbar puncture in patient BII at 26 years of age showed markedly diminished levels of Aβ42 at 34.95 pg/mL (normal range, >190 pg/mL) and elevated phosphorylated tau at 152.3 pg/mL (normal range, <78 pg/mL) consistent with Alzheimer’s disease.

### Ophthalmological findings

**Figure 5:**
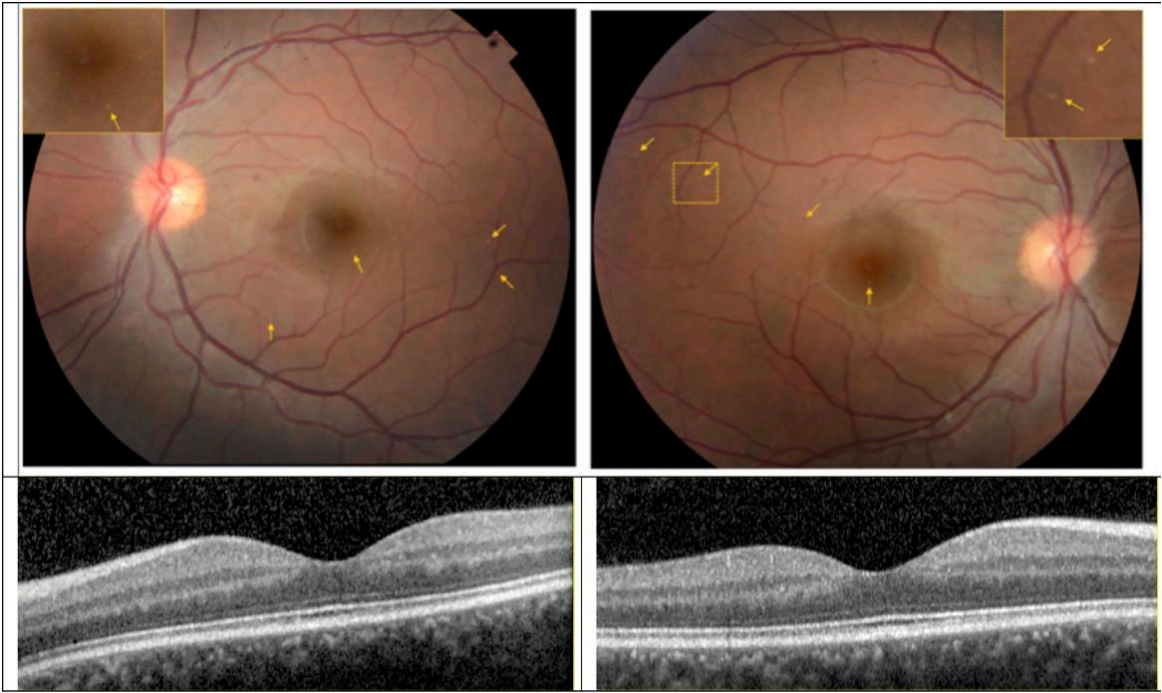
**Colour fundus photograph and optical coherence tomography (OCT) of the left (left panels) and right eyes (right panels) of subject BII.** The optic disc appears with sharp margins and normal colour in both eyes. The retinal vessels are largely unremarkable except for possibly mild tortuosity and focal areas of attenuation of the large calibre retinal arteries in each eye. The fundus pigmentation is notable for small (∼25 microns), focal lesions in each eye some of which are denoted by the small arrows. Inset panels in the fundus photographs demonstrate high magnification of a region with the lesions. OCT sections through the fovea of each are illustrated. There was no evidence of retinal pigment epithelium or intraretinal pathology on any OCT section that corresponded with lesions on fundus photography.

Colour fundus photographs of the posterior pole of BII examined at age 26 were unremarkable except for the appearance of numerous fine, yellow retinal deposits in the fovea and paravascular distribution in each eye (Fig. 5). It is unclear from fundus photographs if the lesions were intraretinal, subretinal or within the retinal pigment epithelium. OCT of the parafoveal macula did not demonstrate any obvious subretinal or intraretinal lesions that corresponded to the yellow deposits on fundus photographs (Figure Section 14/25 OD and OS through fovea). Qualitative analysis of retinal sublayers demonstrated a noticeable increase in the thickness of the outer plexiform layer in the superior quadrant of the fovea in both eyes. Ophthalmological examination of BIII at age 25 was without any unequivocal abnormalities.

### Neuropathologic findings

**Figure 6:**
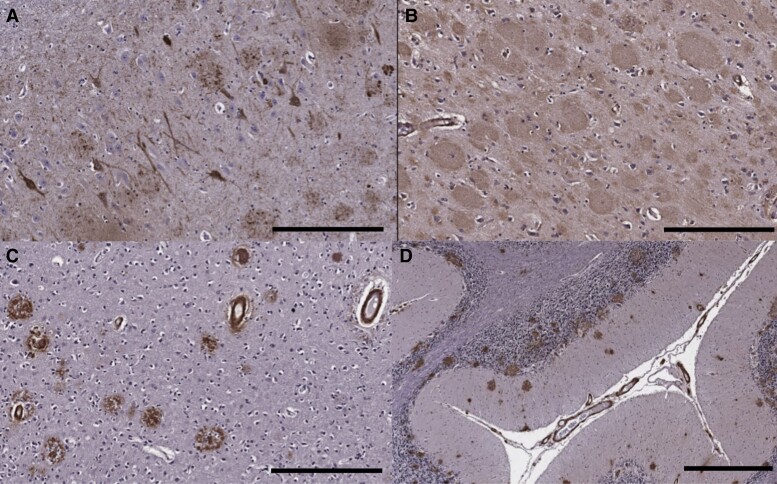
**Immunohistochemistry on neuropathological specimens from the patient with the F388S *PSEN1* mutation.** (**A**) The hippocampus shows abundant neurofibrillary tangles and neuritic plaques on tau (AT8) immunohistochemistry (scale bar = 300 μm). (**B**) Florid amyloid plaques with ‘cotton wool’ morphology in the frontal cortex on Aβ42 immunostain (300 μm). (**D**) Immunohistochemistry for Aβ40 highlights severe CAA in the occipital cortex as well as scattered plaques with dense cores (300 μm). (**D**) Many amyloid plaques as well as CAA seen in the cerebellar cortex on Aβ42 immunostain (scale bar = 600 μm).

Family member BI, who was bed-bound and in assisted living by the age of 27, succumbed to COVID-19 infection at the age of 29. A brain-only autopsy was performed with a post-mortem interval of ∼24 h. The brain weight was 1460 g and showed only minor cerebral cortical atrophy, slightly more pronounced in the occipital lobe. The brainstem appeared smaller than usual. The substantia nigra and locus coeruleus were well-pigmented. Microscopic examination revealed severe Alzheimer’s disease neuropathologic change, A3B3C3 according to NIA-AA guidelines.^19^ Abundant neurofibrillary tangles, neuropil threads and neuritic plaques were seen in the frontal, temporal and occipital cortices and hippocampus, with many neurofibrillary tangles in the hippocampal pyramidal cell layer, on immunohistochemistry for hyperphosphorylated tau (AT8), consistent with Braak and Braak stage VI (Fig. 6A). There were florid senile plaques, many with ‘cotton wool’ morphology that were strongly immunoreactive for Aβ 1–42 but faintly immunoreactive for Aβ 1–40 and with minor neuritic change, spanning the full thickness of the cortices (Fig. 6B). Numerous senile plaques were also present in the hippocampus, basal ganglia, midbrain and cerebellum. There was severe leptomeningeal and cortical CAA, most prominent in the occipital lobes, with both arteriolar and capillary involvement throughout the cerebral and cerebellar cortices (Fig. 6C and D). CAA-associated microangiopathies including vascular hyalinization and occasional microaneurysm formation as well as periarteriolar neuritic change were present in some regions.

**Figure 7:**
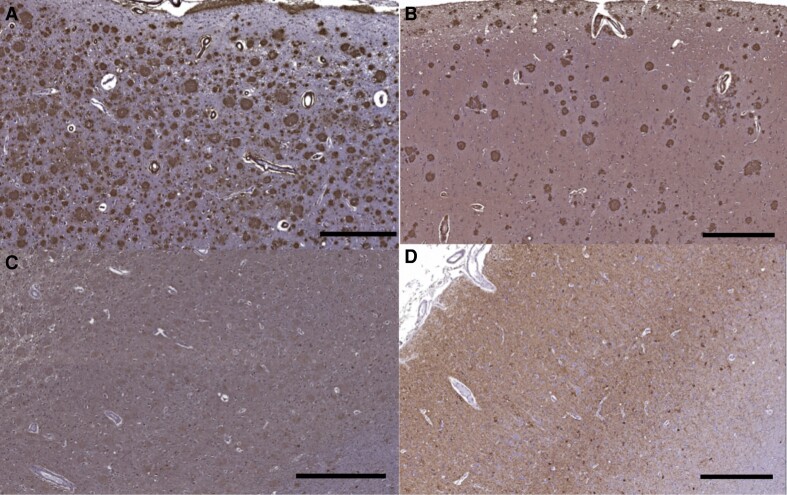
**Immunohistochemistry of motor cortex in the patient with the F388S *PSEN1* mutation and in an autosomal dominant Alzheimer’s disease control without spastic paraparesis.** (**A**) Motor cortex shows amyloid plaques seen with Aβ42 immunostain in the index case with the F388S mutation in *PSEN1* and (**B**) a patient with the V717I mutation in *APP* that does not cause SP (scale bar = 500 μm), (**C**) tau pathology in motor cortex with the F388S mutation (scale bar = 600 μm) and (**D**) with the V717I mutation (scale bar = 500 μm), tau pathology is not obviously more severe in the patient with SP.

**Figure 8:**
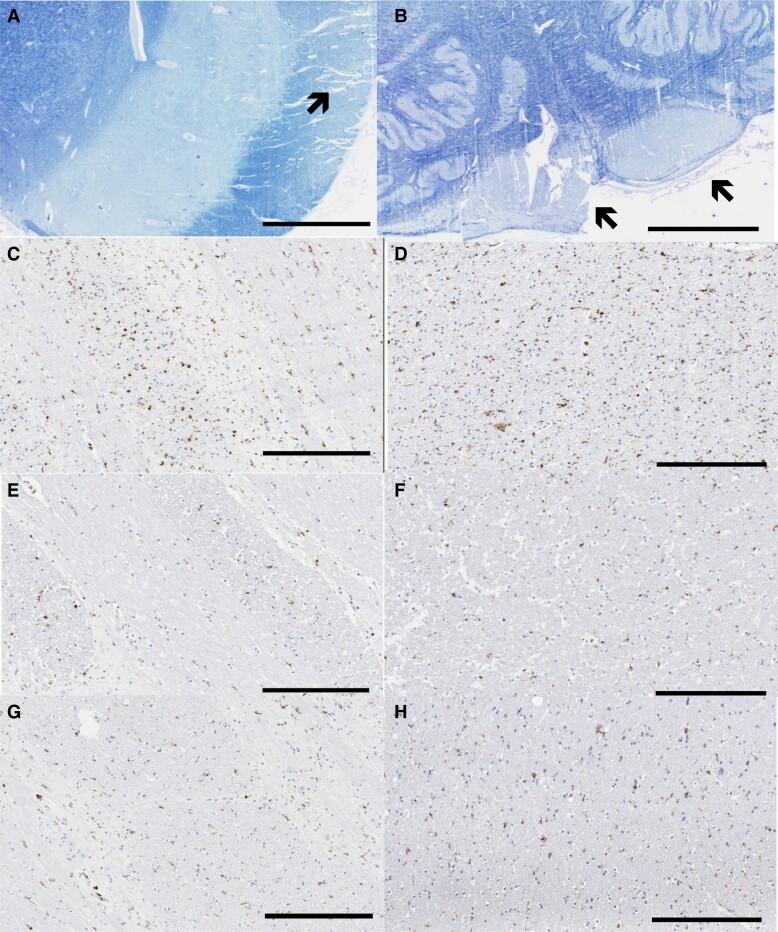
**Degeneration of the corticospinal tracts.** Marked pallor of the cerebral peduncle (indicated by arrow, **A**) and medullary pyramids (**B**) on Luxol fast blue stain (scale bar = 3 mm). CD68 stain in the basis pontis in the F388S mutation carrier with SP (**C**), a control without neurodegenerative disease (**E**) and a person with sporadic Alzheimer’s disease of late onset (**G**). CD68 stains in the cerebral peduncle in the midbrain in the F388S mutation carrier (**D**), a control without neurodegenerative disease (**F**) and a person with sporadic Alzheimer’s disease of late onset (**H**) (scale bars = 300 μm). Note the increased microglial activity in the patient with SP.

Motor cortex showed Aβ42 pathology that appeared qualitatively more severe than that seen due to an autosomal dominant Alzheimer’s disease mutation that does not cause SP (V717I mutation in *APP* though tau pathology was not obviously different), (Fig. 7A–D). Histologic sections of the brainstem showed marked pallor of the corticospinal tracts bilaterally on Luxol fast blue stain with microgliosis on CD68 immunohistochemistry that was greater than in a control without neurodegenerative disease (Fig. 8).

### Aβ profile assay results

**Figure 9:**
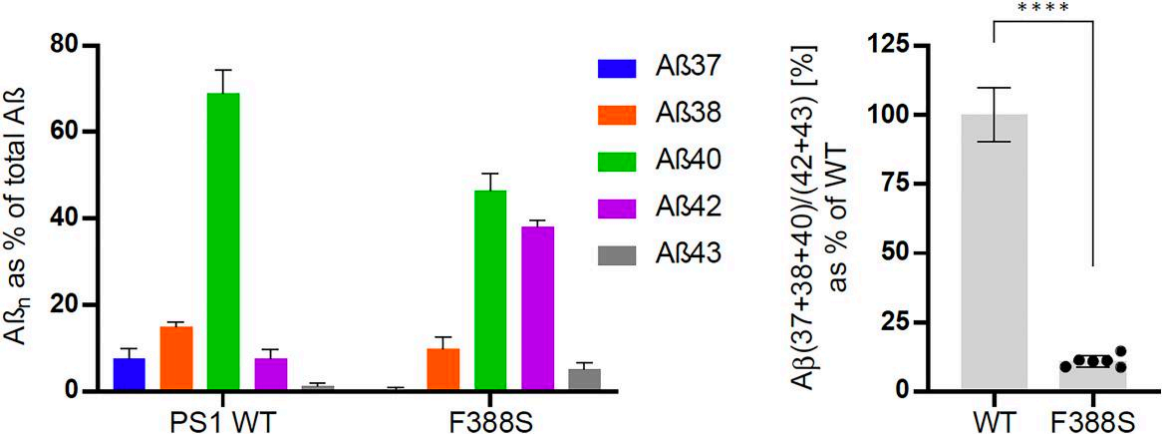
**Aβ profiles generated by wild type and F388S *PSEN1* mutant mouse embryonic fibroblast cell lines.** On the left are Aβ profiles and on the right are the Aβ (37 + 38 + 40)/(42 + 43) ratios as per cent of wild type. Data are represented as mean ± SD in six independent experiments. Statistical significance was assessed by unpaired Student *t*-test (****, *P* < 0.0001).

Measurement of levels of Aβ species produced in the F388S *PSEN1* mouse embryonic fibroblast cell lines showed diminished levels of Aβ37, Aβ38 and Aβ40 and increased levels of Aβ42 and Aβ43 relative to controls (Fig. 9). Using the previously published formula [Aβ (37 + 38 + 40)/(42 + 43)] to calculate an estimated age of disease^21^ onset yields an age of 24.4 years.

## Discussion

In a recent case report and literature review, 13.7% of *PSEN1* mutations were found to cause SP, with 7.5% presenting initially with SP.^2^ We describe a family with a novel *PSEN1* mutation (F388S) causing an extremely young age of autosomal dominant Alzheimer’s disease onset (23 years) that co-segregated with the disease. The heterozygous variant found in *SPG7* (Ala510Val) is unlikely to be relevant as it was not found in the affected mother, *SPG7* acts recessively, and the global frequency of this minor allele is reported to be 0.2% (https://www.ncbi.nlm.nih.gov/clinvar/variation/42016/). The clinical presentation was dominated by progressive pyramidal spasticity with loss of independent ambulation by age 27 with cognition being relatively spared until later in the disease. The mother had been diagnosed with multiple sclerosis, her son with hereditary spastic paraplegia, and the diagnosis of Alzheimer’s disease was not considered until whole-genome sequencing revealed the *PSEN1* variant. Notably at age 26, the younger brother, despite being unable to communicate verbally, still arranged his appointments independently via text message.

The presence of cerebral amyloidosis and neurofibrillary pathology was confirmed using florbetapir and flortaucipir PET, respectively. The florbetapir scan showed diffuse amyloid deposition in the cortex but with an atypically high signal in the basal ganglia, cerebellum and occipital lobe. Though increased deposition in the basal ganglia and other subcortical structures has been previously described in autosomal dominant Alzheimer’s disease,^24–27^ possibly representing extensive cotton wool plaques, disproportionate involvement of the occipital lobe has not been previously noted to our knowledge. Unfortunately, florbetapir scans were not performed on other study participants so no quantitative comparisons can be made.

The flortaucipir signal was also atypical with relative sparing of the medial temporal lobe^28^ though the precuneus was involved as is commonly described in varied forms of Alzheimer’s disease. The occipital lobe was also heavily affected by tau pathology. The flortaucipir signal was non-significantly elevated in the precentral gyrus and paracentral lobule relative to matched carriers of autosomal dominant Alzheimer’s disease mutations that do not feature substantial spasticity. Carriers of the A431E mutation in *PSEN1*, which variably features SP though at a more advanced age,^22, 23^ had intermediate levels of flortaucipir binding in the paracentral gyrus. Across our population, the strongest correlations between flortaucipir SUVs and Ashworth score, a measure of leg spasticity, were found in the precentral and paracentral lobules (Pearson’s *r*’s ranging from 0.778 to 0.814). A prior report of a *PSEN1* mutation presenting with SP also beginning at age 23 also found increased tau deposition by flortaucipir PET in motor cortex contralateral to the most affected leg.^29^ This correlation suggests that loss of neuronal integrity associated with neurofibrillary pathology there may underlie SP. However, no unusual degree of tau pathology was appreciable on neuropathological examination.

Cotton wool plaques have been associated with autosomal dominant Alzheimer’s disease with SP due to *PSEN1* mutations,^30^ although they can occasionally be observed in autosomal dominant Alzheimer’s disease without SP as well as in sporadic Alzheimer’s disease.^31–33^ In the patient that came to autopsy, there were numerous cotton wool plaques, but classic cored and neuritic plaques were also seen. There was also unusually severe CAA with both arteriolar and capillary CAA. Mann *et al*.^34^ have demonstrated increased CAA severity in patients with *PSEN1* mutations beyond codon 200.

Diffusion MRI revealed widespread ultrastructural abnormalities of white matter. Diffusion parameters were significantly and dramatically affected in widespread brain areas in relation to the F388S *PSEN1* mutation, intermediate in association with the A431E *PSEN1* mutation, and lowest with other autosomal dominant Alzheimer’s disease mutations not causing SP. Increased MD was most evident in white matter underlying the precentral gyrus and paracentral lobule and in length of the corticospinal tract where pallor on myelin stains could be appreciated on neuropathological exam. We have previously demonstrated decreased fractional anisotropy in several brain areas among preclinical carriers of autosomal dominant Alzheimer’s disease mutations^35^ though other investigators have not found as robust changes in preclinical disease.^25,36^ As the majority of persons in our initial study had the A431E *PSEN1* mutation in which SP is common and we showed widespread diffusion MRI abnormalities to be present among A431E *PSEN1* mutation carriers with this complication relative to those without,^11^ we hypothesize that these changes underlie SP. The current findings of dramatic diffusion changes associated with the F388S *PSEN1* mutation support this conclusion. These DTI changes also correlated with the degree of spasticity across the study population. As has been previously described in association with SP in association with another *PSEN1* mutation, we found evidence of microglial activation in motor tracts.^37^ Also consistent with our findings, these investigators did not note particularly robust neurofibrillary pathology nor neuronal loss in motor cortex. These findings support a direct effect of *PSEN1* mutations on axonal integrity, rather than anterograde degeneration from loss of cortical projection neurons, despite the relatively high signal in motor cortex seen on flortaucipir PET.

As part of our research protocol, patient BII underwent an ophthalmological evaluation, including fundoscopy, at age 26 when his CDR score was 1.0. Fine yellow retinal lesions were observed on fundoscopy and fundus photography that, though non-specific, were unusual for a young and otherwise healthy man. Though the existence of amyloid plaques in the retina is a topic of controversy, prior studies using flat-mounts of the retina demonstrate amyloid staining in Alzheimer’s disease patients.^38^ Their visibility during life may potentially be enhanced by the prior ingestion of curcumin.^38^ We hypothesize that amyloid deposition in the retina may occur disproportionately in aggressive forms of autosomal dominant Alzheimer’s disease such as that conferred by the F388S mutation.

Notably, as in the current family, the earliest onset autosomal dominant Alzheimer’s disease cases are associated with mutations in the *PSEN1* gene^4^ and are associated with spastic paraplegia in 13.7% of such mutations. Comparison of the mean age of onset among *PSEN1* mutations with (*n* = 36) and without SP at some point in their illness (*n* = 207) in the AlzForum Mutation database (https://www.alzforum.org/mutations/psen-1) demonstrates a lower age of onset among mutations causing SP (36 versus 44 years, *P* < 0.001), suggesting a relationship between SP and particularly aggressive disease. Interestingly, however, when the age of onset of cases presenting initially with SP is compared to those without SP as the first manifestation, a slightly ‘older’ age of onset was noted (40.5 versus 37.3 years of age).^2^ It should also be noted that a case with onset at age 24 (due to the L173W mutation in *PSEN1*^39^) was not reported to be associated with SP. The pathophysiological mechanisms underlying the particularly young onset cases are unclear, as such mutations tend to be distributed throughout the *PSEN1* gene.^4^ It might be speculated that the very young onset in our family is due to the proximity of the affected codon to the critical aspartate residue that comprises the active γ-secretase cleavage site in PS1. However, other mutations in this region do not necessarily lead to a particularly young age of onset. For example, the G384A substitution causes onset around age 35,^40^ and three mutations at codon 386 (F386S, F386I and F386L) have ages of onset of 36–58,^41^ 40^42^ and between 40 and 52,^43^ respectively. The other previously described mutation at codon 388 (F388L) has been demonstrated to increase the production of Aβ42 relative to Aβ40 but has an average age of onset of 43.^44^ Recent evidence showing a correlation between age of onset and degree of altered kinetics in the interaction between γ-secretase and APP conferred by *PSEN1* mutations suggests that increased production of longer length Aβ species may account for differences in the age of onset.^7^ Consistent with a prior publication in which the Aβ (37 + 38 + 40)/(42 + 43) ratio produced *in vivo* accurately predicts the age of symptom onset among *PSEN1* mutations,^21^ such modelling predicts an age of onset of 24.3 years, very close to the 23 year age of onset consistently seen in this family. However, it is also possible that affects on γ-secretase cleavage of substrates besides APP could contribute to a younger age of onset and to SP.^8^

To summarize, we present an in-depth characterization of an extreme form of autosomal dominant Alzheimer’s disease caused by a novel mutation in *PSEN1* with onset in the early 20s presenting with SP that was not diagnosed as Alzheimer’s disease until whole-genome sequencing was performed. The affected brothers had non-significantly disproportionate flortaucipir signal in motor cortex and also in the occipital lobe. Infrastructural changes in white matter only detectable using DTI were robust and significantly more severe than among carriers of another *PSEN1* mutation that more variably causes SP at a later age, which were in turn more severe than among autosomal dominant Alzheimer’s disease mutation carriers without SP. These changes correlated with measures of leg spasticity, potentially suggesting a causative relationship. Consistent with prior autopsied cases of Alzheimer’s disease with SP, cotton wool plaques were present as was rarefaction and microglial activation in the corticospinal tracts in the brainstem. Finally, abnormal retinal lesions were present that may represent amyloid deposits in this aggressive form of Alzheimer’s disease. That the Aβ profiles produced predicted the young age of onset suggests an amyloid-driven aetiology though the link between this and the white matter pathology remains uncertain. Quantitative neuropathological studies comparing persons with and without SP should help define this relationship.

## Abbreviations

Aβ =: amyloid-β
APP =: amyloid precursor protein
CAA =: cerebral amyloid angiopathy
CDR =: Clinical Dementia Rating
DTI =: diffusion tensor imaging
MD =: mean diffusivity
OCT =: optical coherence tomography
SP =: spastic paraparesis
SUVR =: standard uptake value ratio
